# Metabolomic Analysis of the Effects of Canagliflozin on HFpEF Rats and Its Underlying Mechanism

**DOI:** 10.2174/0118715303373321250108174111

**Published:** 2025-01-17

**Authors:** Guorui Zhang, Qingjuan Zuo, Sai Ma, Lili He, Zhongli Wang, Jianlong Zhai, Tingting Zhang, Yan Wang, Yifang Guo

**Affiliations:** 1 Department of Internal Medicine, Hebei Medical University, Shijiazhuang, Hebei, China;; 2 Department of Cardiology, The Third Hospital of Shijiazhuang City Affiliated to Hebei Medical University, Shijiazhuang, Hebei, China;; 3 Department of Geriatric Cardiology, Hebei General Hospital, Shijiazhuang, Hebei, China;; 4 Department of Pain Medicine, Hebei General Hospital, Shijiazhuang, Hebei, China;; 5 Department of Physical Examination Center, Hebei General Hospital, Shijiazhuang, Hebei, China;; 6 Department of Cardiology, Hebei General Hospital, Shijiazhuang, Hebei, China

**Keywords:** Canagliflozin, hypertension, HFpEF, metabonomics, H9C2 cardiomyocytes, energy metabolism

## Abstract

**Background:**

Heart failure with preserved ejection fraction (HFpEF) represents a challenging cardiovascular condition characterized by normal systolic function but impaired diastolic performance. Despite its increasing prevalence, therapeutic options remain limited. This study investigated the metabolic effects of canagliflozin, a sodium-glucose cotransporter 2 (SGLT2) inhibitor, on cardiac function and energy metabolism in HFpEF.

**Methods:**

We established a rat model of HFpEF using Dahl salt-sensitive rats and evaluated three experimental groups: control (A), HFpEF (B), and canagliflozin-treated HFpEF (C). This study carried out comprehensive analyses of cardiac structure and function, metabolomic profiling, and detailed assessment of myocardial energy metabolism, including mitochondrial respiratory capacity and ATP synthesis. Additionally, we validated our findings using H9C2 cardiomyocytes under controlled conditions.

**Results:**

Canagliflozin treatment significantly improved cardiac remodeling markers, including reduced myocardial volume and fibrosis area, while enhancing diastolic function (E/A ratio). Metabolomic analysis revealed normalization of hypermetabolic states, with significant reductions in key metabolites, including L-lysine, D-glucose, and uridine. The treatment restored balance in multiple metabolic pathways, particularly affecting β-alanine metabolism, pyrimidine metabolism, and the citrate cycle. Notably, canagliflozin enhanced mitochondrial respiratory function, increased ATP synthesis, and optimized fatty acid utilization, as evidenced by reduced free fatty acid content.

**Conclusion:**

Our findings demonstrated that canagliflozin exerts cardioprotective effects through multiple metabolic pathways, suggesting its potential as a therapeutic option for HFpEF. The ability of the drug to optimize energy metabolism and improve mitochondrial function represents a novel mechanism for treating this challenging condition.

## 1 INTRODUCTION

Heart failure represents a complex cardiovascular syndrome that poses significant challenges to global health systems and patient outcomes. The recent evolution in our understanding has led to more nuanced classifications of heart failure based on left ventricular ejection fraction (LVEF), including heart failure with reduced ejection fraction (LVEF<40%), mildly reduced ejection fraction (LVEF 41-49%), preserved ejection fraction (HFpEF; LVEF≥50%), and improved ejection fraction [1]. This classification system has particularly highlighted HFpEF as a distinct entity characterized by normal systolic function but impaired diastolic performance [2].

The clinical landscape of HFpEF is further complicated by its frequent association with conditions, such as hypertension, type 2 diabetes mellitus, obesity, and renal dysfunction [3]. Despite intensive research efforts and its growing prevalence, therapeutic options for HFpEF remain limited, resulting in substantial healthcare costs and high mortality rates [4]. This persistent treatment gap has driven the search for novel therapeutic approaches grounded in a deeper understanding of underlying disease mechanisms.

At the heart of HFpEF pathophysiology lies metabolic remodeling, a process that fundamentally alters cardiac energetics before manifesting as structural changes. Recent investigations have revealed that disruptions in mitochondrial function, electron transport chain activity, and energy substrate utilization create a cascade of events that significantly impact cardiac performance [5, 6]. The unique metabolic demands of the heart, being a high-energy-consuming organ with limited storage capacity, make it particularly susceptible to these metabolic perturbations [7, 8].

In this therapeutic landscape, the emergence of sodium-glucose cotransporter 2 inhibitors (SGLT2i) has offered new hope. These agents have demonstrated remarkable efficacy in reducing both cardiovascular mortality and heart failure events [9], though the precise mechanisms underlying their benefits remain a subject of active investigation [10]. The multi-faceted effects of SGLT2 inhibitors, encompassing anti-inflammatory actions, diuretic properties, and metabolic modulation, suggest a complex therapeutic profile particularly relevant to HFpEF treatment [11].

Among the SGLT2 inhibitors, canagliflozin has emerged as a promising candidate for HFpEF treatment, demonstrating beneficial effects beyond its established glucose-lowering properties [12]. However, the specific mechanisms through which canagliflozin influences myocardial metabolism in HFpEF remain incompletely understood. Although previous studies reported some effects of SGLT2 inhibitors on HFpEF, the detailed regulatory mechanisms of canagliflozin on metabolic pathways in HFpEF and how these regulations are related to the improvement of cardiac function are still not fully elucidated. Therefore, our study aimed to fill this knowledge gap by comprehensively analyzing the metabolic effects of canagliflozin in HFpEF through a detailed study of cardiac energy dynamics. We utilized a well-characterized salt-sensitive rat model to investigate how canagliflozin influences cardiac structure, function, and metabolism. Through detailed metabolomic profiling and mitochondrial function analysis, our research provides novel insights into both HFpEF pathophysiology and the therapeutic potential of SGLT2 inhibitors, potentially opening new avenues for targeted treatment strategies.

## 2 MATERIALS AND METHODS

### 2.1 Study Animals and Sample Collection

All animal procedures were conducted in accordance with the Guide for the Care and Use of Laboratory Animals (National Institutes of Health) and received approval from the Animal Care and Management Committee of Hebei General Hospital (permit number: SYXK(JI)2016-0006). Thirty-six specific pathogen-free male Dahl salt-sensitive rats (age 7-8 weeks, weight 230 ± 20 g) were sourced from Beijing Vital River Laboratory Animal Technology Co., Ltd. (animal certificate no. 1100112011029010). The animals were housed in the Clinical Research Center of Hebei General Hospital under controlled environmental conditions (temperature 22 ± 2°C, humidity 55 ± 5%).

We randomly allocated the rats into three experimental groups of twelve animals each: normal control (Group A), HFpEF model (Group B), and canagliflozin treatment (Group C). Animals were housed in three per cage (Tecniplast, Italy, Model 1291H) with free access to food and water. Group A received a 0.3% NaCl diet (Research Diets Inc., D12450B) and normal saline (2 ml/kg/day) *via* gavage. Groups B and C received an 8% NaCl diet (Research Diets Inc., D12469B), with Group C additionally receiving canagliflozin (30 mg/kg/day; Janssen-Cilag International NV, Batch CG392-K1) *via* oral gavage for 12 weeks.

### 2.2 Physiological Monitoring and Sample Collection

Blood pressure measurements were performed weekly using a computerized tail-cuff system (BP-2000, Visitech Systems Inc., SN: BP2K-234). The system was calibrated before each session, and measurements were conducted at the same time of the day to minimize circadian variations. For each animal, we recorded the average of three stable measurements after a five-minute acclimatization period.

Cardiac function was assessed using the Vevo^®^ 2100 imaging system (Fujifilm VisualSonics Inc., Toronto, Canada) equipped with an MS-250 transducer (13-24MHz). Animals were anesthetized using an isoflurane vaporizer (SurgiVet, Model V3000PK) with 4% isoflurane for induction and 1.5-2% for maintenance. We measured left ventricular parameters, including anterior and posterior wall thickness, end-diastolic and end-systolic diameters, and the E/A ratio.

After 12 weeks, following overnight fasting, animals were euthanized using pentobarbital sodium (30 mg/kg, Sigma-Aldrich, P3761) administered intraperitoneally. Blood samples were collected from the abdominal aorta using BD Vacutainer^®^ SSTTM tubes (BD Biosciences, 367814). Hearts were rapidly excised, weighed using an analytical balance (Mettler Toledo, XPE205), and processed for various analyses. A portion of each heart was immediately preserved in 4% paraformaldehyde (Sigma-Aldrich, P6148) for histological studies, while the remaining tissue was either used for immediate mitochondrial analysis or flash-frozen in liquid nitrogen and stored at -80°C (Thermo Scientific, Forma 88000).

### 2.3 Metabolic Cage Studies

Individual metabolic assessments were conducted using specialized metabolic cages (SA104, Jiangsu SANS Biological Technology Co., Ltd., China). Prior to experimental measurements, rats underwent a two-week acclimatization period in the metabolic cages. During this period and throughout the study, we monitored 24-hour water consumption using graduated water bottles (Techniplast, ACBT0262), food intake using precision feeders (Techniplast, 3700M081), and urine output using calibrated collection tubes. The metabolic parameters were recorded daily at 9:00 AM to maintain consistency.

### 2.4 Histological and Immunohistochemistry Analyses

Heart tissue processing began immediately following collection, with careful attention to maintaining tissue integrity. We fixed a 5-mm portion from the apex in a 4% paraformaldehyde solution prepared in phosphate-buffered saline (pH 7.4, Sigma-Aldrich, P3813). After 24-hour fixation at 4°C, tissues underwent dehydration through a graded ethanol series (70%, 80%, 90%, and 100%; Merck, Germany) and xylene clearing before paraffin embedding (Leica EG1150H embedding station).

For morphological evaluation, we prepared 5-μm sections using a rotary microtome (Leica RM2245). Sections underwent hematoxylin and eosin (H&E) staining using Mayer's hematoxylin (Sigma-Aldrich, MHS16) and Eosin Y (Sigma-Aldrich, E4009) following standard protocols. To assess myocardial fibrosis, adjacent sections underwent Masson's trichrome staining using a commercial kit (Sigma-Aldrich, HT15) according to the manufacturer’s specifications.

Stained sections were examined using a Nikon Eclipse Ci-L microscope equipped with a DS-Fi3 camera. For quantitative analysis, we captured images at 200× and 400× magnification using NIS-Elements imaging software (version 5.21). Cardiomyocyte diameter measurements were performed on H&E-stained sections at 400× magnification, with 20 randomly selected cells measured per section using Image-Pro Plus v6.0 software (Media Cybernetics, Inc.).

### 2.5 Biochemical Analysis of Blood Samples

We conducted comprehensive biochemical analyses using both automated systems and specific immunoassays. Blood glucose profiling included fasting blood glucose (FBG) and intraperitoneal glucose tolerance testing (IPGTT). For IPGTT, we administered glucose (2 g/kg body weight; Sigma-Aldrich, G8270) intraperitoneally after a 12-hour fast, with blood glucose measurements at 0, 15, 30, 60, and 120 minutes using a Contour Next glucose meter.

Insulin levels were quantified using a β-cell pancreatic ELISA kit (ExCell Biotech, ER010r) with a sensitivity of 0.1 ng/mL. We calculated the homeostasis model assessment of insulin resistance (HOMA-IR) using the formula: HOMA-IR = FBG × FINS / 22.5. Additional biochemical parameters were measured using a DS-261 automated analyzer (SINNOWA Medical Science and Technology) calibrated daily with manufacturer-supplied standards.

B-type natriuretic peptide (BNP) levels were determined using a specific ELISA kit (CUSABIO, CSB-E07972r) with a detection range of 62.5-4000 pg/mL. All assays were performed in duplicate, with coefficients of variation <10% considered acceptable.

### 2.6 Determination of Myocardial Metabolic Function

Fresh myocardial tissue (approximately 0.1 g) underwent careful processing for mitochondrial isolation. The tissue was first washed in ice-cold PBS (Gibco, 10010023) and homogenized in an isolation buffer using a glass-Teflon homogenizer (Sigma-Aldrich, D9063). The homogenate was processed through differential centrifugation using a Beckman Coulter Optima XPN-100 ultracentrifuge with temperature maintenance at 4°C throughout the procedure.

Mitochondrial respiratory function was assessed using a Clark-type oxygen electrode (Hansatech Instruments, UK) calibrated with air-saturated buffer. We monitored oxygen consumption rates in response to various substrates, including pyruvate/malate (5 mM/2.5 mM) for complex I-linked respiration and succinate (10 mM) with rotenone (2μM) for complex II-linked respiration.

### 2.7 Cell Culture and *In vitro* Studies

H9C2 cardiomyocytes were obtained from Procella Biotechnology Co., Ltd. (Wuhan, China), with authentication confirmed by short tandem repeat profiling. Cells were maintained in Dulbecco's Modified Eagle Medium (DMEM, Gibco, 11995065) supplemented with 10% fetal bovine serum (FBS, Gibco, 10270106) and 1% penicillin-streptomycin (Gibco, 15140122). Culture conditions were maintained at 37°C in a humidified atmosphere containing 5% CO_2_ using a Thermo Scientific™ Heracell™ VIOS 160i incubator.

For experimental procedures, cells were seeded into 96-well plates (Corning, 3596) at a density of 1×10^4^ cells per well. The HFpEF cellular model was established through controlled hypoxia exposure using a specialized hypoxia chamber (Baker Ruskinn InvivO_2_). Cells underwent 12 hours of hypoxia (95% N_2_, 5% CO_2_), followed by 4 hours of reoxygenation. Canagliflozin treatment (10 μmol/L) was administered using a stock solution prepared in DMSO (final DMSO concentration <0.1%).

### 2.8 Metabolomic Analysis using UHPLC-QTOF-MS

Cardiac tissue samples (100 mg) were homogenized in liquid nitrogen using a Retsch MM400 mixer mill. Metabolite extraction was performed using a precise mixture of acetonitrile:methanol:water (2:2:1 v/v/v) containing isotopically labeled internal standards. The extraction process included three cycles of homogenization at 35 Hz for 4 minutes, followed by sonication in an ice-water bath using a Branson Digital Sonifier.

Chromatographic separation was achieved using a Vanquish UHPLC system (Thermo Fisher Scientific) equipped with a UPLC BEH amide column (2.1 × 100 mm, 1.7 μm). The mobile phase consisted of water containing 25 mmol/L ammonium acetate and 25 mmol/L ammonium hydroxide (pH 9.75, Solution A) and acetonitrile (Solution B). The gradient program was carefully optimized to achieve optimal metabolite separation.

Mass spectrometry analysis was performed using a Q Exactive HFX mass spectrometer (Orbitrap MS) operating in both positive and negative ionization modes. Instrument parameters were set as follows: sheath gas flow rate of 30 Arb, auxiliary gas flow rate of 25 Arb, capillary temperature of 350°C, full MS resolution of 60,000, and MS/MS resolution of 7,500.

### 2.9 Statistical Analysis

All statistical analyses were performed using GraphPad Prism software (version 9.5.1). Data normality was assessed using the Shapiro-Wilk test. Normally distributed data are presented as means ± standard deviation. Comparisons between groups were made using one-way ANOVA, followed by Tukey's post-hoc test for multiple comparisons. This is because our data met the assumptions of normality and homogeneity of variance, and Tukey's post-hoc test can effectively control the overall error rate and conduct detailed comparisons between groups under such circumstances. For non-normally distributed data, the Kruskal-Wallis test was carried out, followed by Dunn's post-hoc test, as these methods are suitable for data that do not meet the normality assumption and can more accurately detect differences between groups.

Sample size calculations were performed using G*Power software (version 3.1.9.7) based on preliminary data, with α = 0.05 and β = 0.20 (power = 0.80). For all analyses, *p*-values less than 0.05 were considered statistically significant. Each experiment was performed at least three times independently to ensure reproducibility.

### 2.10 Ethical Approval

All experimental procedures were conducted in accordance with the ethical standards of the institutional and national research committee, adhering to the Declaration of Helsinki principles. The study protocol received approval from the Ethics Committee of Hebei General Hospital (approval number: 2024-LW-089) and complied with accepted principles of medical ethics. In addition, we adhered to the key principles of the ARRIVE guidelines in our *in vivo* experiments, including ensuring animal welfare, reasonable experimental design, and accurate reporting of results, thus ensuring the scientific and standardized nature of the experiment.

## 3 RESULTS

### 3.1 Canagliflozin Attenuates Cardiac Remodeling in HFpEF Rats

**Fig. (1):**
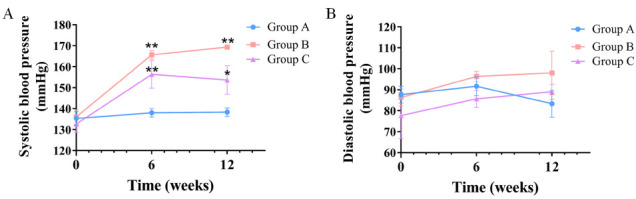
The blood pressure of Dahl salt-sensitive rats at 0, 6, and 12 weeks. (**A**) Systolic blood pressure of Dahl salt-sensitive rats between the three groups. (**B**) Diastolic blood pressure of Dahl salt-sensitive rats between the three groups (*p < 0.05, **p < 0.01).

Our investigation of blood pressure dynamics revealed compelling evidence of the therapeutic potential of canagliflozin in HFpEF. The high-salt diet effectively induced hypertension in our model, with systolic blood pressure rising markedly in Group B after 6 weeks (Fig. **1A**). Intriguingly, while canagliflozin treatment did not immediately normalize blood pressure, we observed a gradual but significant reduction in systolic pressure by week 12 in Group C. The diastolic blood pressure showed similar patterns, though the treatment effect was more subtle (Fig. **1B**).

**Fig. (2):**
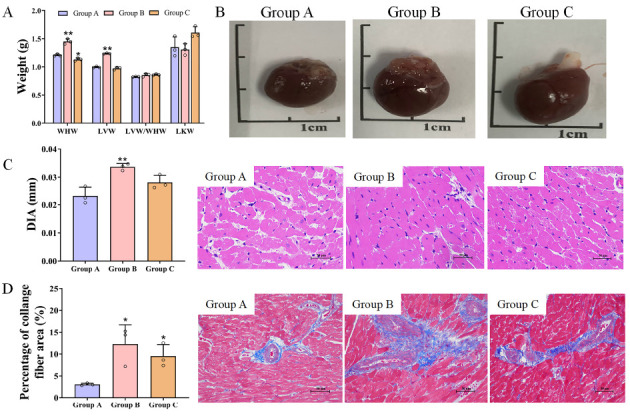
(**A**) Heart and kidney weights between the three groups. The whole heart weight (WHW) and left ventricular weight (LVW) in Group B were significantly higher than those in groups A and C. (**B**) Group B had the largest left ventricle size, which could be reduced in Group C. (**C**) Diameter (DIA) of cardiomyocytes of three group. The DIA of cardiomyocytes was reduced with canagliflozin treatment. (**D**) Masson staining showed the area of myocardial fibrosis in the three groups. The drug treatment reduced the fibrotic area in the rat model of heart failure (*p < 0.05, **p < 0.01).

The impact of canagliflozin on cardiac structure was particularly striking. Analysis of heart morphometry revealed that the high-salt diet induced substantial cardiac enlargement in Group B, with both whole-heart weight and left ventricular weight significantly exceeding those of the control group (1.52 ± 0.09 g *vs.* 1.20 ± 0.09 g, *p* < 0.01). Remarkably, canagliflozin treatment prevented this pathological growth, maintaining heart weights close to control levels (Fig. **2A**). This effect was visually apparent in the gross examination of the left ventricle, where the enlarged chambers in Group B showed notable reduction with treatment (Fig. **2B**).

Perhaps most compelling were our histological findings. Microscopic examination revealed that cardiomyocytes in the HFpEF model had undergone significant hypertrophy, with their diameter increasing by nearly 70% compared to controls (0.034 ± 0.002 mm *vs.* 0.020 ± 0.003 mm). Canagliflozin treatment substantially attenuated this cellular enlargement (Fig. **2C**). The development of fibrosis, a hallmark of pathological cardiac remodeling, showed an equally impressive response to treatment. Masson's trichrome staining revealed extensive fibrotic areas in untreated HFpEF rats, but canagliflozin intervention reduced this scarring by approximately 30% (Fig. **2D**).

### 3.2 Preservation of Cardiac Function under Canagliflozin Treatment

**Fig. (3):**
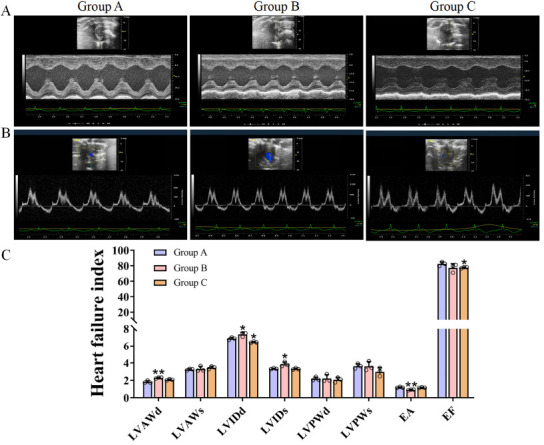
Effect of canagliflozin treatment on cardiac ultrasound results. (**A**, **B**) echocardiography of three groups. (**C**) Echocardiography heart failure index of three groups. The EA values of the rats with heart failure were significantly increased after canagliflozin treatment (*p < 0.05, **p < 0.01).

Our echocardiographic investigations yielded particularly interesting insights into cardiac function. While conventional parameters remained relatively stable across groups, we discovered a remarkable improvement in diastolic function with canagliflozin treatment. The E/A ratio, a critical indicator of diastolic performance, showed significant normalization in treated animals (1.19 ± 0.08 *vs.* 0.93 ± 0.04, *p* = 0.02) (Figs. **3A**-**C**). This finding suggests that canagliflozin not only prevents structural remodeling but also preserves functional cardiac dynamics.

### 3.3 Canagliflozin Modulates Oxidative Stress Response

**Fig. (4):**
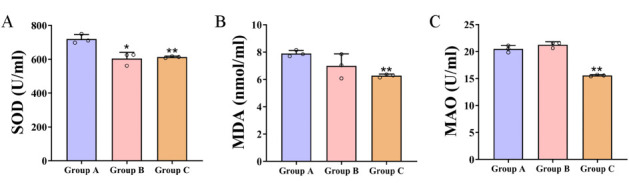
Effect of canagliflozin treatment on the plasma oxidation/antioxidant system. (**A**) The level of superoxide dismutase (SOD) in plasma. (**B**) The levels of MDA in plasma. Canagliflozin decreased the levels of MDA in hypertensive heart failure rats. (**C**) The levels of MAO in plasma. Canagliflozin decreased the levels of MAO in hypertensive heart failure rats (*p < 0.05, **p < 0.01).

Our investigation into oxidative stress markers revealed the nuanced effects of canagliflozin treatment. While superoxide dismutase (SOD) levels remained relatively stable across experimental groups (Fig. **4A**), we observed significant changes in other key oxidative markers. Notably, malondialdehyde (MDA) levels, a reliable indicator of oxidative damage, showed a marked reduction in canagliflozin-treated animals compared to the HFpEF model group (6.18 ± 0.23 *vs.* 6.69 ± 1.07 nmol/ml, *p* < 0.05) (Fig. **4B**). Even more striking was the reduction in monoamine oxidase (MAO) levels with treatment (15.73 ± 2.41 *vs.* 21.16 ± 1.30 U/ml, *p* < 0.01) (Fig. **4C**), suggesting comprehensive modulation of oxidative stress pathways.

### 3.4 Beneficial Effects on Glucose Metabolism

**Fig. (5):**
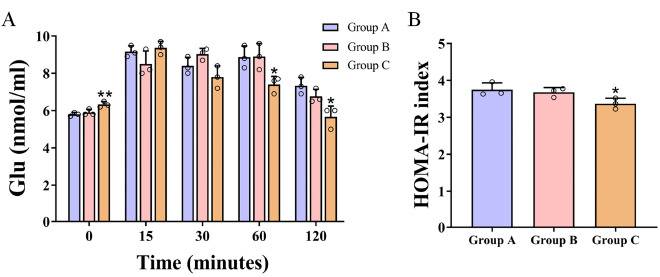
Effect of canagliflozin treatment on blood glucose. (**A**) Fasting and postprandial blood glucose. Canagliflozin can significantly reduce blood glucose at 1 and 2 hours postprandial in rats with hypertensive heart disease. (**B**) HOMA-IR index. Canagliflozin reduced the HOMA-IR index in the rat model of hypertensive heart disease (*p < 0.05, **p < 0.01).

Canagliflozin demonstrated remarkable effects on glucose homeostasis in our HFpEF model. The glucose tolerance testing revealed particularly interesting temporal patterns. While early post-prandial glucose levels (15 and 30 minutes) showed minimal differences between groups, we observed significant improvements at the 1-hour (8.05 ± 1.26 *vs.* 8.65 ± 1.28 mmol/ml, *p* < 0.05) and 2-hour marks (5.63 ± 0.71 *vs.* 6.83 ± 0.76 mmol/ml, *p* < 0.05) in treated animals (Fig. **5A**). Perhaps most significantly, it was found that canagliflozin treatment led to a meaningful reduction in insulin resistance, as evidenced by improved HOMA-IR values (3.16 ± 0.51 *vs.* 3.56 ± 0.49, *p* < 0.05) (Fig. **5B**).

### 3.5 Comprehensive Biochemical Profile Changes

Our analysis of blood biochemistry revealed fascinating patterns of metabolic adaptation. Treatment with canagliflozin induced significant increases in both lactate dehydrogenase and alanine aminotransferase levels (Table **1**), suggesting enhanced metabolic activity. While creatinine levels showed a trend toward improvement with treatment, this effect did not reach statistical significance. Particularly noteworthy was the marked reduction in B-type natriuretic peptide levels in treated animals, indicating improved cardiac stress status.

The urinary biochemical analysis provided additional insights into the mechanisms of action of canagliflozin. We observed substantial decreases in urinary creatinine and protein excretion in treated animals (Table **2**). Most remarkably, the treatment group showed significantly increased urinary glucose excretion, confirming the primary mechanism of the drug while also demonstrating reduced electrolyte excretion.

### 3.6 Metabolomic Insights Reveal Complex Pathway Modulation

**Fig. (6):**
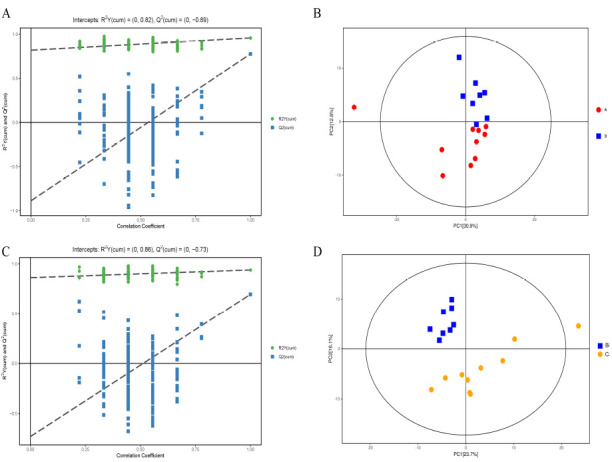
Metabonomics of myocardial tissues. (**A**) Overfitting analysis between Group A and Group B. (**B**) Principal component analysis (PCA) between Group A and Group B. (**C**) Overfitting analysis between Group B and Group C. (**D**) Principal component analysis (PCA) between Group B and Group C.

Our metabolomic analysis uncovered fascinating shifts in cardiac metabolism across experimental groups. The principal component analysis (PCA) revealed distinct metabolic signatures, with a clear separation between control and HFpEF groups, and notably, a shift toward normalization in the canagliflozin-treated group (Figs. **6B** and **D**). Particularly intriguing was the Q2 value progression in our random forest model, which demonstrated robust predictive power without overfitting (Figs. **6A** and **C**), validating the reliability of our metabolomic findings.

**Fig. (7):**
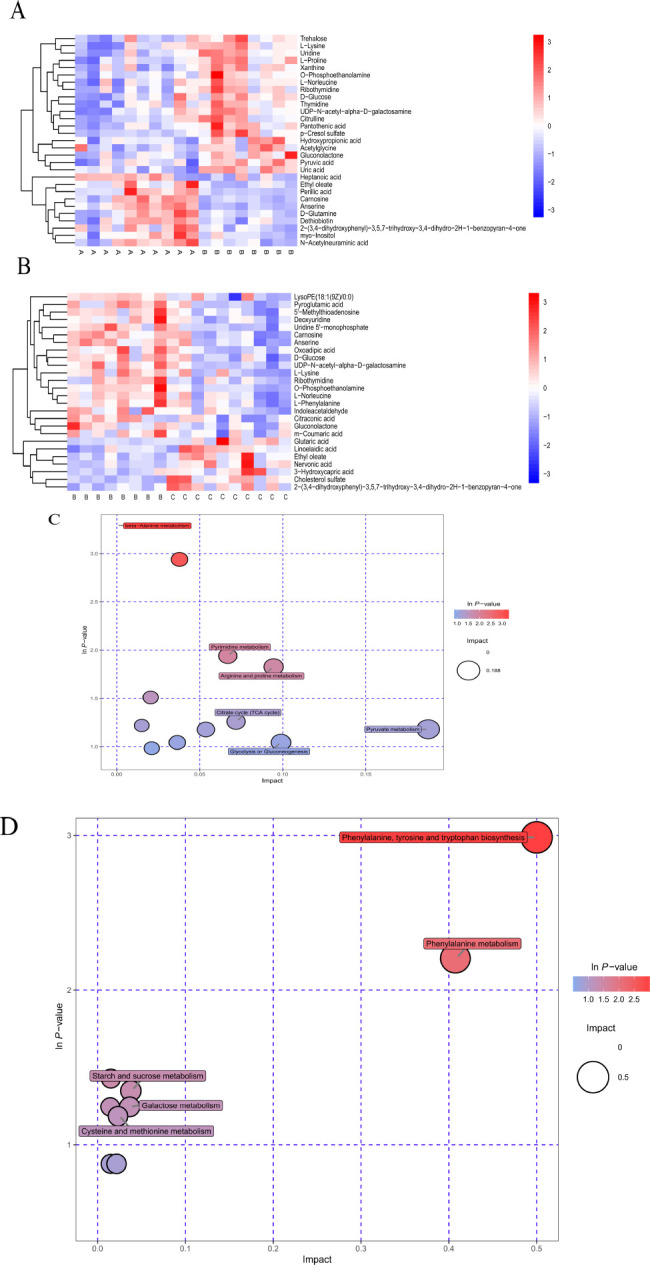
Metabonomics of myocardial tissues. (**A**) Significantly differed compounds in abundance between groups A and B. (**B**) Significantly differed compounds in abundance between groups B and C. (**C**) KEGG pathways of significantly differed compounds between groups A and B. (**D**) KEGG pathways of significantly differed compounds between groups B and C.

The negative ion mode mass spectrometry analysis identified 35 significantly altered metabolites between control and HFpEF groups and 26 between HFpEF and treatment groups. The changes in L-lysine, D-glucose, and uridine levels were particularly striking, suggesting fundamental alterations in energy metabolism pathways. These findings are illustrated in our hierarchical clustering analysis (Figs. **7A** and **B**).

Our pathway analysis revealed that canagliflozin treatment led to significant modulation of several key metabolic processes. We observed the normalization of previously dysregulated pathways involved in β-alanine metabolism, pyrimidine metabolism, and the citrate cycle in treated animals (Figs. **7C** and **D**). This comprehensive metabolic remodeling suggests that the therapeutic effects of canagliflozin extend far beyond its known glucose-lowering properties.

### 3.7 Deep Dive into Myocardial Energy Dynamics

**Fig. (8):**
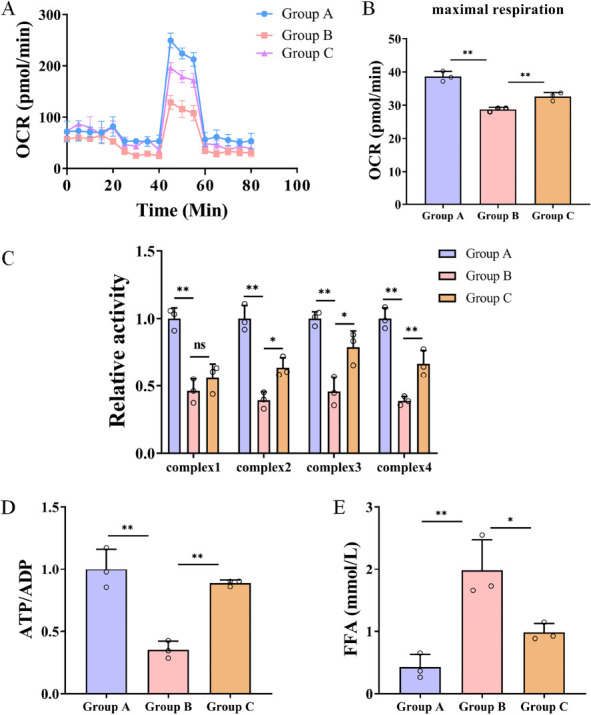
Effects of canagliflozin on mitochondrial function and metabolic parameters in rat myocardial tissues. (**A**) Time course of mitochondrial oxygen consumption rate (OCR). (**B**) Maximal respiration rate (OCR). (**C**) Relative activity of mitochondrial complexes. (**D**) ATP/ADP ratio. (**E**) Free Fatty Acid (FFA) content (*p < 0.05, **p < 0.01).

Our investigation of myocardial energy metabolism yielded particularly compelling results. The oxygen consumption rate (OCR) measurements revealed a significant decline in mitochondrial function in HFpEF rats, which showed marked improvement with canagliflozin treatment (Figs. **8A** and **B**). The activity patterns of respiratory chain complexes revealed a similarly intriguing narrative, with all complexes showing reduced function in untreated HFpEF rats but significant recovery with treatment (Fig. **8C**).

The ATP/ADP ratio findings were especially noteworthy. We observed a substantial decrease in energy availability in HFpEF hearts, but canagliflozin treatment effectively restored cellular energetics (Fig. **8D**). Complementing these findings, free fatty acid levels showed significant reduction with treatment (Fig. **8E**), suggesting improved metabolic efficiency.

**Fig. (9):**
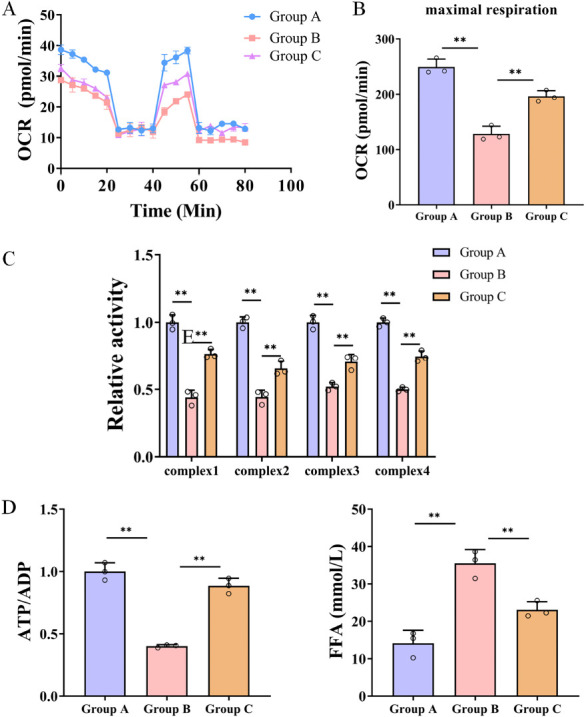
Effects of canagliflozin on mitochondrial function and metabolic parameters in H9C2 cells. (**A**) Time course of mitochondrial oxygen consumption rate (OCR). (**B**) Maximal respiration rate (OCR). (**C**) Relative activity of mitochondrial complexes. (**D**) ATP/ADP ratio. (**E**) Free Fatty Acid (FFA) content (*p < 0.05, **p < 0.01).

Most excitingly, our *in vitro* studies using H9C2 cells provided robust validation of these findings. The cellular model demonstrated remarkably similar patterns in OCR, ATP/ADP ratios, and free fatty acid content (Figs. **9A**-**E**), strengthening the direct cardiac effects of canagliflozin. These parallel findings between our animal model and cell culture studies provide compelling evidence for the therapeutic mechanism of canagliflozin in HFpEF.

## 4 DISCUSSION

SGLT2 inhibitors have emerged as a promising therapeutic class, demonstrating significant reductions in cardiovascular mortality and complications in patients with type 2 diabetes [13]. Our findings extend this understanding by revealing powerful cardioprotective effects in HFpEF, with canagliflozin showing particular promise in this challenging condition. The observed improvements in cardiac function and metabolism provide compelling evidence for its therapeutic potential [14]. A notable finding from our study is the impact of canagliflozin on cardiac remodeling. Our data align with recent clinical observations showing that SGLT2 inhibitors can effectively reduce cardiovascular events and heart failure-related hospitalizations in both diabetic and non-diabetic populations [15, 16]. The improvements in cardiometabolic function and vascular properties suggest multiple beneficial mechanisms [17].

The renal protective effects observed deserve particular attention. By inhibiting glucose and sodium reabsorption in the proximal renal tubules, canagliflozin appears to enhance tubuloglomerular feedback, leading to improved glomerular function [18]. This renal protection, combined with reduced plasma volume and blood pressure, contributes to the overall cardiovascular benefits [17, 18]. Our findings support previous observations that canagliflozin can improve BNP levels and reduce pericardial fat volume, potentially accelerating the reversal of cardiac remodeling [19]. The antioxidant and anti-inflammatory properties revealed in our study provide new mechanistic insights. Building on previous research, we found that canagliflozin improves vascular inflammation and atherosclerosis through multiple pathways [20-22]. Recent studies have revealed that canagliflozin activates new signaling pathways or molecular mechanisms, such as the Nrf2/ARE pathway, to enhance the antioxidant defense system [23]. These effects are believed to be mediated by the reduced expression of vascular adhesion molecules and inflammatory cytokines, as demonstrated by Xu *et al.* (2018), who reported that canagliflozin exerts anti-inflammatory effects by inhibiting intracellular glucose metabolism and promoting autophagy in immune cells [24]. Hasan *et al.* (2020) further supported this by demonstrating that canagliflozin attenuates isoprenaline-induced cardiac oxidative stress by stimulating multiple antioxidant and anti-inflammatory signaling pathways [25]. These findings indicate that the antioxidant mechanism of canagliflozin may be more complex and diverse than previously understood, offering new directions for future research.

While we observed modest changes in SOD levels, our findings regarding oxidative stress markers align with previous studies showing the ability of canagliflozin to maintain SOD activity and upregulate its expression [26]. The reduction in MDA levels is particularly significant, as elevated MDA has been associated with increased oxidative stress in HFpEF models [27]. The renal protective effects observed in our study complement existing evidence showing the benefits of SGLT2 inhibitors in reducing proteinuria and stabilizing serum creatinine levels [28]. Our metabolomic findings regarding the protective role of L-lysine against high glucose-induced damage add to our understanding of cellular protection mechanisms [29].

The improvements in mitochondrial function observed are particularly significant given that mitochondrial dysfunction is central to myocardial metabolic disorders [30]. The enhanced activity of respiratory chain complexes I-IV suggests improved oxidative phosphorylation and ATP generation [31, 32], addressing a fundamental aspect of heart failure pathophysiology [33]. Our findings regarding metabolic substrate utilization provide new insights into cardiac energetics. The ability of the heart to utilize various energy substrates, including fatty acids, glucose, and ketone bodies, is crucial for maintaining function. The normalization of fatty acid oxidation and reduction in lactate accumulation observed suggest that canagliflozin helps optimize cardiac energy metabolism.

These findings have important implications for our understanding and treatment of HFpEF. The complex pathophysiology of HFpEF, involving both structural and metabolic alterations, has historically made it resistant to conventional heart failure therapies. The metabolic improvements observed, particularly in mitochondrial function and energy efficiency, address a fundamental aspect of HFpEF pathophysiology that has been difficult to target with existing treatments. The restoration of proper energy utilization patterns, coupled with improved oxidative stress management, suggests that SGLT2 inhibitors might help break the vicious cycle of metabolic dysfunction and cardiac deterioration often seen in HFpEF patients.

Our findings regarding the time course of therapeutic effects have significant clinical relevance. The gradual improvement in cardiac parameters suggests that early intervention with SGLT2 inhibitors might be beneficial in preventing or slowing the progression of HFpEF. The parallel findings between our animal model and cellular studies strengthen the translational potential of our research, suggesting that the beneficial effects of canagliflozin are likely to be reproducible in human patients. However, we acknowledge several important limitations of our study. In terms of model selection, although the Dahl salt-sensitive rat model used can effectively simulate some characteristics of HFpEF, there are certain limitations. For example, compared with human HFpEF, there are differences in the physiological structure and metabolic characteristics of the cardiovascular system in rats, which may lead to incomplete consistency in drug responses. Moreover, the method of inducing HFpEF in the model (high-salt diet) may not fully cover all the etiologies and pathophysiological processes of human HFpEF, which may affect the extrapolation of the research results. Future studies should consider using multiple models or models that are closer to the characteristics of human diseases to more comprehensively evaluate the role of canagliflozin in HFpEF. In addition, the relatively short duration of our study is also a limitation. Short-term studies cannot observe the long-term potential effects of the drug, including possible drug resistance, late adverse reactions, and long-term stable effects on cardiac structure and function. Therefore, long-term follow-up studies are needed to more accurately evaluate the safety and effectiveness of canagliflozin in the treatment of HFpEF.

## CONCLUSION

Our comprehensive investigation demonstrated that canagliflozin provides significant cardioprotective effects in HFpEF through multiple interconnected mechanisms. The ability of the drug to improve cardiac remodeling, reduce myocardial fibrosis, and normalize hypermetabolic states represents a promising therapeutic approach. The enhancement of energy metabolism through improved mitochondrial function and optimized metabolic pathways provides compelling evidence for SGLT2 inhibitors as a valuable treatment option in HFpEF.

From a clinical practice perspective, our findings suggest that canagliflozin may be a potentially effective drug option for the treatment of HFpEF. Clinicians may consider including canagliflozin in the treatment regimen for HFpEF patients and closely monitor cardiac function, metabolic indicators, and potential adverse reactions. For future research, our findings lay the foundation for further studies on the optimal dosage, treatment timing, and combination therapy strategies of canagliflozin in HFpEF. In addition, large-scale clinical trials are needed to verify the effectiveness and safety of our findings in human patients and to further explore the detailed molecular mechanisms by which canagliflozin improves cardiac function and metabolism, providing a theoretical basis for the development of more effective treatment methods for HFpEF.

## Figures and Tables

**Table 1:** Biochemical parameters of blood samples.

-	Group A (n=8)	Group B (n=6)	Group C (n=8)
ALB (g/L)	30.11 ± 1.56	27.96 ± 1.93	27.58 ± 2.82
LDH (U/L)	473.8 ± 56.03	358.8 ± 31.26	433.2 ± 48.01
CHE (U/L)	26.02 ± 6.44	26.44 ± 3.62	25.90 ± 6.96
CK (U/ml)	1.03 ± 0.12	0.94 ± 0.07	0.99 ± 0.14
ALT (U/L)	7.23 ± 2.10	6.86 ± 1.84	10.32 ± 1.83
AST (U/L)	9.30 ± 1.74	8.85 ± 1.33	8.49 ± 0.94
Cr (μmol/L)	27.48 ± 4.07	40.06 ± 7.57	37.14 ± 7.21
TC (mmol/L)	2.54 ± 0.39	3.47 ± 0.97	3.78 ± 0.75
LDL (mmol/L)	1.43 ± 0.15	1.99 ± 0.42	1.97 ± 0.33
K (mmol/L)	3.84 ± 0.18	3.95 ± 0.14	3.89 ± 0.27
Na (mmol/L)	171.91 ± 13.39	168.80 ± 15.28	170.22 ± 7.62
BNP (pg/ml)	245.28 ± 45.57	329.12 ± 15.92^*^	220.37 ± 75.55^#^

**Note: ** The data are presented as the mean±standard deviation. *p < 0.05 *versus* Group A. ^#^*p* < 0.05 *versus* Group C.

**Table 2:** Biochemical parameters of the urine samples.

-	Group A (n=6)	Group B (n=6)	Group C (n=6)
Cr (μmol/L)	6559 ± 4348^*^	10068 ± 3573	1625 ± 245.4^#^
Urea protein (g/L)	0.39 ± 0.22^*^	1.82 ± 0.45	0.90 ± 0.40^#^
K (mmol/L)	67.55 ± 41.90^*^	84.07 ± 27.04	29.62 ± 5.30^#^
Na (mmol/L)	21.01 ± 5.75^*^	154.4 ± 76.16	128.6 ± 19.02^#^
Cl (mmol/L)	70.07 ± 33.47^*^	133.30 ± 42.50	125.4 ± 6.81^#^
Urea glucose (mmol/L)	0.05 ± 0.02^*^	0.13 ± 0.04	50.20 ± 8.09^#^

**Note: ** The data are presented as the mean±standard deviation. *p < 0.05 *versus* Group A. ^#^*p* < 0.05 *versus* Group C.

## Data Availability

The complete dataset can be made available upon request from the corresponding author (yifangguo@hebmu.edu.cn).

## LIST OF ABBREVIATIONS

ALT: Alanine Aminotransferase
ATP/ADP: Adenosine Tri/Diphosphate
BUN: Blood Urea Nitrogen
CK: Creatine Kinase
EF: Ejection Fraction
E/A: Early to Late Diastolic Filling Velocity Ratio
FBG: Fasting Blood Glucose
FFA: Free Fatty Acid
FINS: Fasting Insulin
HFpEF: Heart Failure with Preserved Ejection Fraction
HOMA-IR: Homeostasis Model Assessment of Insulin Resistance
LDL: Low-Density Lipoprotein
LKW: Left Kidney Weight
LVW: Left Ventricular Weight
LVAWd/s: Left Ventricular Anterior Wall Thickness in Diastole/Systole
LVIDd/s: Left Ventricular Internal Diameter in Diastole/Systole
LVPWd/s: Left Ventricular Posterior Wall in Diastole/Systole
LVEF: Left Ventricular Ejection Fraction
MAO: Monoamine Oxidase
MDA: Malondialdehyde
SGLT-2is: Sodium-Glucose Cotransporter 2 Inhibitors
SOD: Superoxide Dismutase
TC: Total Cholesterol
T2DM: Type 2 Diabetes Mellitus
WHW: Whole-Heart Weight

## DECLARATION

This manuscript was submitted as a pre-print at https://www.researchsquare.com/article/rs-1886973/v.
